# Effects of Coexistence Hypertension and Type II Diabetes on Heart Rate Variability and Cardiorespiratory Fitness

**DOI:** 10.5935/abc.20180105

**Published:** 2018-07

**Authors:** Daniela Bassi, Ramona Cabiddu, Renata G. Mendes, Natália Tossini, Vivian M. Arakelian, Flávia C. R. Caruso, José C. Bonjorno Júnior, Ross Arena, Audrey Borghi-Silva

**Affiliations:** 1 Departamento de Fisioterapia, Universidade Ceuma, São Luís, MA - Brazil; 2 Departamento de Fisioterapia, Universidade Federal de São Carlos, São Carlos, SP - Brazil; 3 Departamento de Fisioterapia, Universidade Nove de Julho, São Paulo, SP - Brazil; 4 Departamento de Medicina, Universidade Federal de São Carlos, São Carlos, SP – Brazil; 5 Departamento de Fisioterapia, Universidade de Illinois em Chicago, Chicago, IL – EUA

**Keywords:** Hypertension/prevalence, Diabetes Mellitus,Type 2, Cardiovascular Diseases, Risk Factors, Autonomic Nervous System, Heart Rate

## Abstract

**Background:**

Type 2 diabetes Mellitus (T2DM) is associated with cardiac autonomic dysfunction, which is an independent predictor of mortality in chronic diseases. However, whether the coexistence of systemic arterial hypertension (HTN) with DMT2 alters cardiac autonomic modulation remains unknown.

**Objective:**

To evaluate the influence of HTN on cardiac autonomic modulation and cardiorespiratory fitness in subjects with DMT2.

**Methods:**

60 patients of both genders were evaluated and allocated to two groups: DMT2 patients (n = 32; 51 ± 7.5 years old) and DMT2 + HTN patients (n = 28; 51 ± 6.9 years old). RR intervals were obtained during rest in supine position. Linear and nonlinear indices of heart rate variability (HRV) were computed using Kubios HRV software. Pulmonary gas exchange was measured breath-by-breath, using a portable telemetric system during maximal incremental exercise testing on a cycle ergometer. Statistical analysis included Shapiro-Wilk test followed by Student’s t Test, Pearson correlation and linear regression.

**Results:**

We found that patients in the DMT2+HTN group showed lower values of mean RR intervals (801.1 *vs* 871.5 ms), Shannon entropy (3 *vs* 3.2) and fractal dimension SD 1 (9.5 vs 14.5), when contrasted with patients in the DMT2 group. Negative correlations were found between some HRV nonlinear indices and exercise capacity indices.

**Conclusion:**

HTN negatively affects the cardiac autonomic function in diabetic patients, who are already prone to develop autonomic dysfunction. Strategies are need to improve cardiac autonomic functionality in this population.

## Introduction

The prevalence of hypertension in patients with type 2 diabetes mellitus (T2DM) is up to three times higher than in patients without T2DM.^1^ The coexistence of hypertension and diabetes significantly increases the probability of developing cardiovascular disease (CVD).^2^

The harmful association of these two conditions may cause deleterious effects on the cardiovascular system, accelerating the atherosclerosis process involved in both T2DM and hypertension.^3^ In addition, it is well known that cardiac autonomic neuropathy (CAN), resulting from damage to the autonomic nerve fibers that innervate the heart and blood vessels, is a serious complication of T2DM^4^ and systemic arterial hypertension (HTN).^5^

The autonomic nervous system plays a significant role in the circulatory system and in blood pressure regulation.^6^ Damage to the nerve fibers that innervate the heart and blood vessels leads to abnormalities in heart rate (HR) control and vascular dynamics.^7^ Heart rate variability (HRV) analysis is a widely used tool to assess the cardiac autonomic regulation.^8^ HRV is commonly analyzed using linear models, such as time domain and spectral analysis; however, non-linear methodologies have been recently proposed as novel tools to investigate the complexity of HR dynamics.^9^

It has been widely documented that reduced HRV is associated with various pathological conditions, including CVDs, such as hypertension^10^ and diabetes.^11^ However, despite the evidence that HRV is reduced in the presence of one of these conditions, it remains unknown whether HRV is altered in the coexistence of T2DM and HTN.

Additionally, it is well established that exercise capacity, which is a strong predictor of cardiovascular and overall mortality,^12^ is reduced in patients with T2DM compared with non-diabetic subjects ^13^ as well as hypertensive patients.^14^ Although the causes of reduced exercise capacity in these populations are unknown, cardiac autonomic dysfunction may play an important role in the development of heart disease in diabetic patients leading to impaired exercise capacity.^15^

Recently, new variables derived from the cardiopulmonary exercise test (CPET), such as circulatory power (CP) and ventilatory power (VP) have been used for the clinical evaluation of heart failure patients as important markers of exercise limitation^16^. These indices could provide a potentially valuable measure of cardiopulmonary function in the coexistence of TM2DM and HTN.

Considering this knowledge gap, the primary objective of the present study was to assess the cardiac autonomic modulation in T2DM patients with and without HTN. The secondary objective was to verify if HRV indices are correlated with exercise capacity in these patients.

We hypothesized that patients affected by DMT2 and HTN would have an altered cardiac autonomic control when compared with diabetics and that there would be a correlation between HRV indexes and exercise capacity.

## Methods

### Design

The present investigation is a cross sectional study.

#### Participants

A total of 60 patients (mean age ± SD = 51 ± 7 years; 42 male and 18 female) diagnosed with T2DM, followed at the cardiovascular outpatient clinic of the Federal University of Sao Carlos (UFSCar), agreed to participate in the study. Patients were divided into two groups according to the presence or not of HTN: 1) DMT2 (n = 32; 20 males and 12 female) and 2) T2DM + HTN (n = 28; 20 males and 8 female). Duration of DMT2 and HTN was recorded, based on the date of diagnosis self-reported by patients. The experimental procedures were performed in the UFSCar Cardiopulmonary Physiotherapy Laboratory.

Inclusion criteria for both groups consisted of age between 40 and 60 years and clinically diagnosed DMT2 – based on fast glycemia and hemoglobin A1c (HbA1c) values, according to current guidelines – currently under hypoglycemics and clinically stable for at least 6 months. All patients were sedentary (self-reported). In the DMT2 + HTN group, diabetic subjects had clinical diagnosis of HTN and were under hypoglycemic and antihypertensive therapy. Exclusion criteria consisted of a history consistent with coronary heart disease or other concomitant respiratory diseases.

#### RR interval recording

The RR intervals were recorded continuously using a Polar S810i telemetry system (Polar Electro Oy, Kempele, Finland) at a sampling rate of 500Hz, and these data were used to derive the HRV indices. Each subject rested for 10 minutes before the initiation of data collection to ensure HR stabilization. The RR interval signal was continuously recorded for 10 minutes, while the patient rested in supine position, breathing spontaneously. Participants were instructed not to speak unnecessarily during the evaluation to avoid HR signal interference.

#### HRV analysis

The RR interval signals were transferred to a microcomputer and reviewed by visual inspection by an independent examiner to verify the quality of the signals and detect any abnormalities. Segments which presented any abnormalities were discarded. The data were transferred to Kubios HRV analysis software (MATLAB, version 2 beta, Kuopio, Finland) and a stable and free of artifacts series of 256 sequential RR intervals was selected and analyzed. To analyze the tachograms, a multivariate approach was followed, which allows for a comprehensive assessment of the cardiac autonomic function.

The nonlinear dynamic properties of HRV were analyzed by calculating approximate entropy (ApEn),^17^ correlation dimension (CD)^18^ and Poincaré plots^19^. ApEn quantifies the regularity of a time series and represents a simple index of the overall complexity and predictability of the signal. High ApEn values indicate high irregularity, while smaller values indicate a more regular signal. Thus, higher ApEn values reflect better health and function.^17^ The CD index represents a measure of the dimensionality of the space occupied by the state vectors or the number of the degrees of freedom of a time series, also referred to as fractal dimension. A higher CD reflects more degrees of freedom of the cardiac sinoatrial node and, therefore, a greater range of possible adaptive responses to internal or external stimuli in an ever-changing environment.^20^

Poincaré plots were built for each RR interval series and the following two descriptors were computed: (i) SD1 – the standard deviation measuring the dispersion of points perpendicular to the line-of-identity. This parameter is usually interpreted as a measure of short-term HRV, which is mainly influenced by respiratory sinus arrhythmia (parasympathetic modulation); and (ii) SD2 – the standard deviation measuring the dispersion of points along the identity line, which is interpreted as a measure of both short- and long-term overall HRV. Shannon entropy (SE) was computed to quantify the degree of complexity of the distribution of the signals samples.^21^

A set of time domain HRV parameters were calculated, including: (i) mean and standard deviation of RR intervals (SD RR), in ms; (ii) square root of the mean squared differences of successive RR intervals (RMSSD), in ms; and (iii) geometrical parameters, including the integral of the RR interval histogram divided by the height of the histogram (RR tri index) and the baseline width of the histogram (TINN), in ms. A spectral analysis was performed on the tachograms, in order to calculate the signal spectral power in the frequency band between 0.03 Hz and 0.14 Hz (low-frequency [LF] band) and in the frequency band between 0.15 Hz and 0.4 Hz (high-frequency [HF] band), both expressed in normalized units.^22^ STD RR represents a global index of HRV and reflects all the cyclic components responsible for variability in the recording period; RMSSD reflects alterations in autonomic tone that are predominantly vagally mediated; the geometrical HRV indices are an estimate of the overall HRV.^23^ However, reference values for these parameters, available in the literature, were obtained in healthy subjects aged from 40 to 60 years –rMSSD from 33.39 to 28.77 (ms) for male and from 30 to 25.80 (ms) for female; HFnu from 22.85 to 24.51 for male and from 27.74 to 27.94 female; LFnu from 77.07 to 75.49 for male and from 72.26 to 72.06 for female; LF/HF from 3.36 to 3.08 for male and from 2.60 to 2.58 female. Reference values for nonlinear variables are also available only for the same age – SD1 from 24.01 to 20.56 for male and from 21.55 to 18.44 (ms) for female and SD2 from 198.61 to 185.20 for male and from 176.15 to 165.41 (ms) for female.^24^

#### Laboratorial exams

Blood samples were obtained after an overnight fast. HbA1c was measured in a central laboratory by anion-exchange high-performance liquid chromatography (Variant II, Bio Rad, Berkeley, California), coupled with a fluorescence detector method certified by the National Glycohemoglobin Standardization Program.^25^

Insulin resistance was evaluated by HOMA–IR using the following formula: (fasting plasma glucose [mg/dL] x fasting plasma insulin [µU/mL] / 22.5).^25^ Fasting plasma glucose was measured by an enzymatic method using an AU 680® (Beckman Couter, Suarlée, Namur, Belgium) and fasting plasma insulin was measured by a chemiluminescent assay (UniCel® DxI 800, Pasadena, California, USA). Total cholesterol (total-C), low-density lipoprotein cholesterol (LDL-C), high-density lipoprotein cholesterol (HDL-C) and triglycerides were measured by an enzymatic method using the AU 680® (Beckman Couter, Suarlée, Namur, Belgium). The Brazilian Society of Diabetes criteria for metabolic control were used as reference values – HbA1c 7% or 53 mmol/mol and fasting plasma glucose < 110 mg/dL.^26^

#### Cardiopulmonary exercise testing (CPET)

A symptom-limited incremental exercise test was performed on a cycle ergometer (Recumbent Corival of MedGraphics - Minnesota, USA.). Gas exchange and ventilatory variables were recorded during the test using a calibrated computer-based exercise system (Metabolic analyzer System Greenhouse telemetry module for field studies Oxycon-Mobile, Jaeger, Hoechberg, Germany).

The day before the CPET, subjects were taken to the experimental room for familiarization with the procedures and equipment to be used.

All subjects were evaluated in the morning to avoid circadian influences on their physiological responses. All subjects were instructed to: (i) avoid caffeinated and alcoholic beverages or any other stimulants (drinks, foods or medications) the night before and the day of data collection; and (ii) not to perform activities requiring moderate-to-heavy physical exertion on the day before data collection. The tests were carried out under controlled relative air humidity and temperature conditions. Before the CPET, the exercise protocol was described to each subject by a member of our group.^27^

Peak VO_2_ was defined as the highest VO_2_ value during the last 15 seconds of exercise.^28^ Fifteen second averaged ventilation (V_E_) and carbon dioxide production (VCO_2_) data, obtained from the initiation of exercise to exercise peak, were input into Microsoft Excel, Microsoft Corp., Bellevue, WA, USA).

#### Outcome measures

**Primary outcome:** The primary outcome measures were the HRV indices, able to detect abnormalities in the cardiac autonomic system regulation.

**Secondary outcome:** As a secondary outcome measure, the exercise capacity was assessed by CP and VP, both of which have been showed to serve as a surrogate predictor of mortality and prognosis.^16^

### Statistical analysis

Data are reported as mean ± SD. All data were verified for the assumptions of normality, and comparisons between groups (T2DM *vs* T2DM+HTN) were performed using unpaired t tests. The categorical variables were presented in percentage (absolute number) and the comparisons between the groups of these variables were performed by means of the chi-square test. Statistical analyses were performed using Statistica 5.5 (StatSoft Inc., Tulsa, USA).

Pearson’s product moment correlation coefficient was used to examine the relationship between linear and nonlinear indices and cardiorespiratory variables. The magnitude of the correlations was determined considering the following classification scheme for r-values ≤ 0.35 low or weak; r = 0.36 ≤ 0.67 moderate; r ≥ 0.68 strong or high; r ≥ 0.9 very high; r = 1 perfect.^29^ The probability of a type I error was set at 5% for all tests (α = 0.05).

## Results

### Subject characteristics

A total of 60 patients were evaluated over a 1-year period. Table 1 shows demographic, anthropometric and clinical characteristics of subjects in the two groups (DMT2 and DMT2+HTN).

**Table 1:** Patients demographic, anthropometric and clinical characteristics

Variables	DMT2 (n = 32)	DMT2+HTN (n = 28)	p value
Gender (males/females)	20/12	20/8	0.464
Age (years)	51 ± 7.5	51 ± 6.9	0.660
Weight (kg)	79.3 ± 9.6	86.2 ± 14*	0.033
Height (m)	1.7 ± 0.1	1.7 ± 0.1	0.450
BMI (kg/m^2^)	28.5 ± 4.4	31 ± 3.8*	0.031
Duration DMT2 (years)	5.7 ± 5.3	6.6 ± 6.4	0.334
Duration HTN (years)	-	3 ± 2.6	-
SBP (mmHg)	129 ± 16	140 ± 20	0.021
DBP (mmHg)	87 ±7	94 ± 12	0.011
**Medications**			
**Antiglycemics - % (n)**			
Biguanides	87.5 (28)	75 (21)	0.312
Sulfonylureas	50 (16)	57.1 (16)	0.613
DPP-4 inhibitors	6.2 (2)	-	-
**Antihypertensive- % (n)**			
ARBII	-	50 (14)	-
Diuretics	-	25 (7)	-
ACE I inhibitors	-	21.4 (6)	-
Renin Inhibitors	-	10.7 (3)	-
β-blocker	-	7.1 (2)	-
Risk factors - % (n)			
Smoking	-	-	-
CAD family history	21.88 (7)	25 (7)	1.000
Sedentarism	100 (32)	100 (28)	1.000
Dyslipidemia	43.75 (14)	46.43 (13)	1.000
**Laboratory exams**			
HbA1c (%)	8 ± 2.14	8.7 ± 1.6	0.394
Insulin (µU/mL)	12 ± 8	19.1 ± 12.5*	0.010
Fasting glucose (mg/dL)	160 ± 69.4	164.6 ± 50.7	0.774
QUICKI	0.34 ± 0.07	0.29 ± 0.02*	0.011
HOMA-IR	4 ± 4	8 ± 6.6*	0.020

Data are expressed as mean ± standard deviation. DMT2: type 2 diabetes Mellitus HTN: arterial hypertension; BMI: body mass index; SBP: systolic blood pressure; DBP: diastolic blood pressure (reference values according to the Brazilian Society of Diabetes criteria; DPP4: dipeptidyl peptidase-4; ARBII: angiotensin II receptor antagonists; ACE I: angiotensin converting enzyme I inhibitor; CAD: coronary artery disease; HbA1c: glycated hemoglobin; QUICKI: quantitative insulin sensitivity check index; HOMA-IR: homeostatic model assessment insulin resistance.

* p<0.05, unpaired Student's t-test or chi-square test.

There were no significant differences between groups in baseline characteristics (age, height, and duration of T2DM). However, BMI was higher in the group of patients with both diseases (p = 0.03). However, no other body composition measurements were performed in order to better characterize the body status. There were no significant differences regarding other risk factors for CVD and oral hypoglycemic medications. Additionally, insulin and HOMA-IR were significantly higher in T2DM + HTN when compared to T2DM, indicating higher insulin resistance. There were no significant differences for fasting plasma glucose, total-C, LDL-C, HDL-C and HbA1c. The HRV indices are presented in Table 2. Mean values of RR intervals and the nonlinear indices SD1, Shannon entropy and ApEn were significantly lower in T2DM +HTN when compared to T2DM. 

**Table 2:** Linear and non-linear HRV indices for both groups in resting conditions

Variables	DMT2 (n = 32)	DMT2+HTN (n = 28)	p value
**Linear**			
Mean RR intervals (ms)	871.5 ± 105.8	801.1 ± 89.0*	0.010
RMSSD (ms)	17.9 ± 11.1	21.2 ± 15.2	0.358
STD RR	29.3 ± 21.5	31.5 ± 23.2	0.718
LF (nu)	66.3 ± 19.8	59.7 ± 22.9	0.247
HF (nu)	33.7 ± 19.8	40.3 ± 22.9	0.241
TINN	110.5 ± 59.8	121.3 ± 67.5	0.523
RR Tri	5.5 ± 2.6	7.1 ± 4.5	0.082
**Nonlinear**			
SD1	14.5 ± 8.2	9.5 ± 4.4*	0.021
SD2	40.4 ± 20.0	43.0 ± 23.1	0.662
SE	3.2 ± 0.3	3.0 ± 0.3*	0.012
ApEn	14.5 ± 8.2	9.5 ± 4.4*	0.021
SampEn	1.4 ± 0.3	1.5 ± 0.3	0.601
CD	1.2 ± 1.3	1.6 ± 1.6	0.271

Data are expressed as Mean ± SD. HRV: heart rate variability; RMSSD: square root of the mean squared differences of successive RR intervals; STD RR: standard deviation of RR; LF nu: normalized unit in the low frequency band; HF nu: normalized unit in the high frequency band; TINN: baseline width of the RR intervals histogram; RR tri: integral of the RR interval histogram divided by the height of the histogram; SD: standard deviation of instantaneous RR interval variability, SE: Shannon Entropy; ApEn: approximate entropy; SampEn: sample entropy, CD: correlation dimension.

* p < 0.05, unpaired Student's t-test.

### Cardiopulmonary exercise test

Table 3 shows the comparison between groups in relation to peak variables obtained during the CPET. Compared with the T2DM group, T2DM+HTN had significantly higher values of systolic blood pressure (SBP) and diastolic blood pressure (DBP) at rest (p = 0.02).

**Table 3:** Cardiopulmonary exercise testing responses

Variables	T2DM (n = 32)	T2DM+HTN (n = 28)	p value
VO_2_ (ml.Kg^-1^.min^-1^)	22.6 ± 7.5	20.4 ± 3.5	0.18
VCO_2_ (mL.min^-1^)	2126.7 ± 673.5	2186.8 ± 510.3	0.72
V_E_ (L.min^-1^)	63.9 ± 19.7	69.5 ± 15.7	0.26
RER	1.2 ± 0.1	1.2 ± 0.1	0.59
V_E_/VCO_2_ slope	28.4 ± 4.6	29.9 ± 4.6	0.27
CP (mmHg.ml.kg^-1^ min^-1^)	4902.7 ± 2004.9	4642.3 ± 1157.1	0.58
VP (mmHg)	1.4 ± 0.5	1.5 ± 0.4	0.26
SBP rest (mmHg)	130.03 ± 16.08	137.6 ± 17.4	0.10
DBP rest (mmHg)	86.7 ± 7.6	92.7 ± 11.2‡	0.02
SBP peak (mmHg)	209 ± 32.1	225.4 ± 24.6‡	0.04
DBP peak (mmHg)	100.38 ± 16.35	104.1 ± 16.4	0.41
Workload (watts)	125.4 ± 37.4	126.5 ± 36.5	0.92

Data are expressed as Mean ± SD.

‡ Unpaired Student's t-test. T2DM: type 2 diabetes mellitus; HTN: hypertension; VO_2_: oxygen uptake; VCO_2_: carbon dioxide production; RER: respiratory exchange ratio; VE/VCO_2_ slope: minute ventilation/carbon dioxide output relationship from the beginning of exercise to peak exercise; CP: circulatory power; VP: ventilator power; SBP: systolic blood pressure, DBP: diastolic blood pressure.

**Figure 1:**
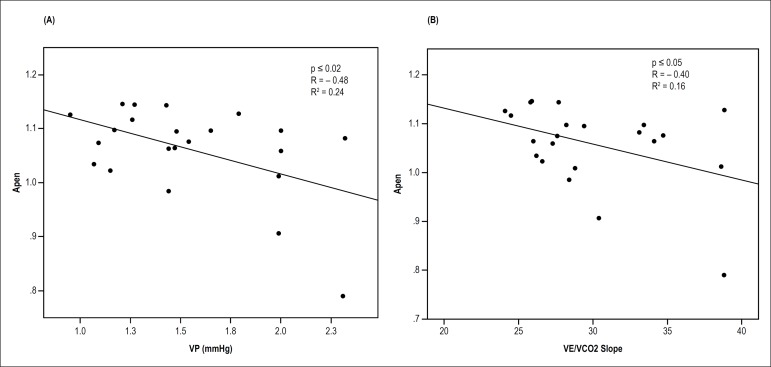
Significant and inverse relationship of approximate entropy (ApEn) with ventilatory power (VP) (A) and minute ventilation/carbon dioxide production ratio (V_E_/VCO_2_) slope (B) in response to peak intensity exercise in patients with type 2 diabetes.

**Figure 2:**
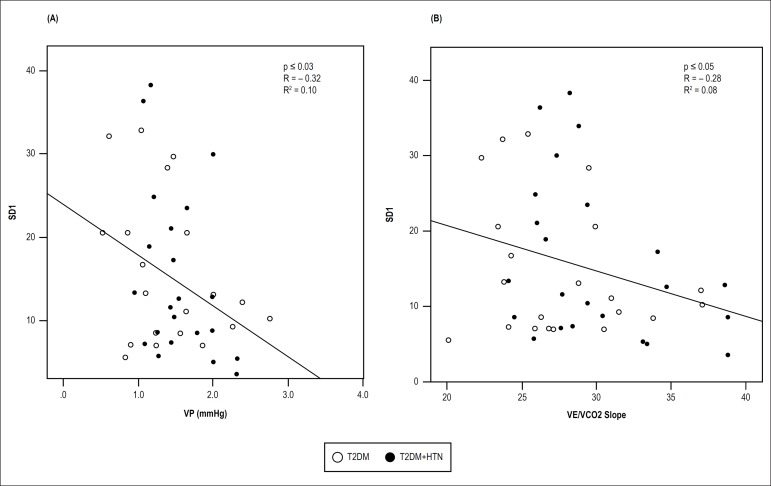
Significant and inverse relationship between SD1 and (A) VP (A) and between SD1 and (B) V_E_/VCO_2_ slope in response to peak intensity exercise in patients with diabetes mellitus type 2 and hypertension (TD2DM+HTN) (●) and in patients with type 2 diabetes mellitus (TD2DM) (°).

Considering only the DMT2 group, we found that ApEn influenced the slope (R^2^ = -0.40, p < 0.05) and the VP (R^2^ = -0.48, p < 0.02) (Figure 1). Finally, when we considered both T2DM and T2DM + HTN groups, we found that the nonlinear indices influenced the VP (R^2^ = -0.10, p < 0.03) and the V_E_/VCO_2_ slope (R^2^ = -0.08, p < 0.05) (Figure 2).

Maximum workload was no different between groups, as well as VO_2_, VCO_2_, respiratory exchange ratio (RER), slope, CP and VP. Stepwise regression analysis was performed to determine the possible influence of HRV indices on CPET variables of interest, which was observed with three of the variables, affected by risk factors – slope was influenced by SD1 (interaction effects: R^2^ = -0.28, p < 0.005) and VP (R^2^ = -0.32, p < 0.03), when both groups considered together.

## Discussion

### Summary of findings

The main findings of the present study are: (i) individuals with DMT2 associated with HTN, even when controlled, presented with a greater impairment in linear and nonlinear HR dynamics compared to those with only DMT2; (ii) novel CPET derived parameters, confirming our hypothesis. To our knowledge, this is the first study to address nonlinear HR dynamics in this specific population. The findings of the present study stress the clinical importance of early evaluation of the cardiac nervous system functionality, once the association between T2DM and HTN alters the cardiac autonomic modulation.

### Relevance of the present study

This is the first study, to our knowledge, to assess linear and nonlinear HRV dynamics in the coexistence of HTN and T2DM. Previous studies have reported cardiac autonomic dysfunction in diabetic subjects and in hypertensive subjects;^30^ this study is relevant, as it showed that there is a simultaneous influence of HTN and DMT2 on nonlinear HRV indexes and on novel CPET derived parameters. In addition, VP and CP, indices that combine parameters of CPET with systemic hemodynamics during exercise represent important physiologic measurements related to the ability to respond to aerobic exertion synergistically. In the present study these indices were shown as important markers of cardiocirculatory limitation to exercise in DMT2 and HTN.

### Effects of the coexistence of DMT2 and HTN on linear and nonlinear HRV dynamics

HRV is reduced in patients with DMT2^31^ as well as in patients with HTN^32^ and its reduction is associated with poor cardiovascular prognosis.^33^ Autonomic imbalance may be a final common pathway to increased morbidity and mortality in the presence of various conditions, including CVD.^34^

Although time and frequency-domain HRV parameters have been shown to be more sensitive in a previous study,^35^ in the present study we did not find significant alterations in these parameters. Roy and Ghatak^36^ in their study with diabetic type 1 patients diagnosed ≥ 5 years earlier, showed that HRV spectral indices were better indicators of the prevalence of CAN than cardiovascular reflex tests.^36^ Meanwhile, the use of HRV spectral analysis only to diagnose CAN should be carefully considered, since previous studies^30,37^ showed low reproducibility of HRV assessment by spectral analysis. The presence of CAN is closely associated with macrovascular complications, mortality due to fatal cardiac arrhythmia, severe hypoglycemia, and sudden death.^38^

However, nonlinear indices have been shown to be better than conventional methods for identifying subtle changes in cardiac autonomic modulation in various pathological conditions such as cardiovascular artery disease.^39^ Nonlinear analysis has provided new insight into the HRV dynamics in various physiological and pathophysiological conditions, providing additional prognostic and analytical information to conventional approaches.^40^ In the currently study, nonlinear indices were found to be reduced in the DMT2+HTN group when compared to the DMT2 group. Additionally, we observed that nonlinear indices of HRV were more sensitive in detecting differences in the autonomic impairment between patients with diabetes and patients with diabetes associated to HTN. ApEn and SE indicated changes that suggest that the coexistence of both diseases is associated to reduced complexity.^41^ In the same way, Roy and Ghatak^36^ showed that nonlinear analytical methods were effective to find differences in HRV patterns between diabetic patients and healthy matched controls. Recently, our group verified that patients with DMT2 with poor glycemic control are more susceptible to poor autonomic nervous control of HR, demonstrated by linear and nonlinear indices.^31^ However, the present study is the first to analyze the coexistence of HTN and DMT2 by means of linear and nonlinear HRV analysis.

 The Diabetes Control and Complications Trial (DCCT) showed that glycemic control can reduce the incidence of CAN.^42^ Previous studies evidenced that a reduction around 11% in the HbA1c improved HRV in patients with type 1 diabetes.^43^

Additionally, Vinik et al.,^4^ showed that the CAN prevalence and mortality rates were higher among individuals with DMT2, probably because of the longer duration of glycemic abnormalities before diagnosis. Our findings showed that, even after a short period from the DMT2 diagnosis, both groups demonstrate poor glycemic control, which might negatively affect HRV and, consequently, increase the patients’ cardiovascular risk.

### Effects of the coexistence of HTN and DMT2 on CPET

CPET represents an easy and non-invasive way to obtain information on the impairment of exercise capacity and of cardiopulmonary fitness.^44^ Ugur-Altun et al.^45^ demonstrated a negative correlation between insulin resistance and peak exercise capacity in diabetic patients. Interestingly, in our study we could not find any differences between groups in peak exercise capacity, maybe because both groups had poor glycemic control, as showed by HbA1c, even though the DMT2+HTN group has shown higher insulin resistance than the DMT2 group.

CP, which is related to the cardiac output and the mean arterial blood pressure at peak exercise, is considered a more powerful predictor of mortality than peak oxygen consumption.^46^ In our study, we have not found differences in CP and VP between groups; however, negative correlations were shown of CP and VP with nonlinear indices of HRV. Castello-Simões et al.^16^ studied patients with CVD (without heart failure) and demonstrated that both CP and VP might hold value as screening tools in assessing not only functional significance but also exercise tolerance, as the impairment of autonomic nervous modulation is related to reduced CP and VP.

The present study has some limitations that need to be stated. First, some relevant information, including DMT2 and HTN diagnostic date and physical activity status were self-reported by the patients and this could introduce a recall bias. Moreover, only the BMI was used to characterize the patients’ body type. However, in order to provide a complete description, other body composition measurements should be considered. Secondly, in the present study a control group comprised of individuals without diabetes mellitus or arterial hypertension could be better clarify the potential influence of these risk factors on HRV indices.

## Conclusion

In summary, cardiac autonomic alteration in the coexistence of DMT2 and HTN was observed when compared to matched DMT2 patients. In addition, the alteration of nonlinear HRV dynamics observed in resting conditions may have negative consequences on these patients’ cardiopulmonary and cardiocirculatory responses.
